# Asthma Management in Pregnancy

**DOI:** 10.1371/journal.pone.0060247

**Published:** 2013-04-04

**Authors:** Rachel A. Charlton, Annie Hutchison, Kourtney J. Davis, Corinne S. de Vries

**Affiliations:** 1 Department of Pharmacy and Pharmacology, University of Bath, Bath, United Kingdom; 2 GlaxoSmithKline, Worldwide Epidemiology, Wavre, Belgium; The University of Adelaide, Australia

## Abstract

**Background:**

Asthma is common during pregnancy, however research is limited regarding the extent and timing of changes in asthma management associated with pregnancy.

**Objective:**

To determine the prevalence of asthma during pregnancy and identify changes in treatment and asthma exacerbation rates associated with pregnancy, while controlling for seasonal influences.

**Methods:**

Pregnant women with asthma were identified from the UK General Practice Research Database between 2000 and 2008. For each woman asthma medication prescribed during the study period was identified; for each product combination the British Thoracic Society medication-defined asthma treatment step was identified. Asthma exacerbations were identified during pregnancy and in the corresponding 12 months prior. Analyses of changes in asthma treatment and exacerbation rates during pregnancy relative to the corresponding period 12 months prior, to control for seasonality, were stratified by trimester and asthma treatment intensity level.

**Results:**

The prevalence of treated asthma in pregnancies resulting in a delivery was 8.3%. From 14,141 pregnancies, in 12,828 women with asthma, 68.4% received prescriptions for a short-acting β_2_-agonist and 41.2% for inhaled corticosteroids; 76.5% were managed with asthma treatment Step 1 or 2. Poor persistence to inhaled corticosteroids, defined as a gap of up to 60 days between prescriptions, was common. In 45.0% of pregnancies, an increase in average treatment step was observed whereas in 25.6% the treatment step decreased. Treatment intensity remained the same in 29.5% of pregnancies. Exacerbations occurred in 4.8% of pregnancies compared to 5.9% in the same season the year before (p<0.001).

**Conclusion:**

Exacerbation rates during pregnancy were slightly lower than in the year before. However, treatment patterns and exacerbation rates in this study suggest asthma control during pregnancy is variable, and women may require close monitoring especially in those with evidence of poor control before pregnancy.

## Introduction

Estimates from published studies suggest that asthma affects between 3–14% of pregnancies [1]–[4] and asthma medicines are commonly used during pregnancy. There are also a number of physiological and mechanical changes during pregnancy that might cause either improvement or worsening (including exacerbations) of asthma symptoms. [5]–[7] A systematic review and meta-analysis of 14 studies from before 1990, evaluating the course of asthma during pregnancy, concluded that approximately one third of women experienced an improvement in their symptoms during pregnancy, one third experienced a worsening and one third stayed the same. [8] It has also been shown that changes in asthma severity occurring during pregnancy often revert back to pre-pregnancy levels within three months of delivery. [9] Exacerbations are most likely to occur during the second and third pregnancy trimesters with a peak at six months gestation. [10]–[11] Although some studies have suggested anecdotally that women with severe asthma are more likely to experience exacerbations during pregnancy than women with mild asthma, [6] the relationship with pre-pregnancy asthma severity has not been evaluated systematically with methods to control for seasonal differences. Insight in these matters will inform women with asthma who want to become pregnant and their clinicians regarding anticipated changes in disease activity in pregnancy and associated needs for alterations in disease management.

This study aimed to determine the prevalence of asthma during pregnancy and to investigate any association between pregnancy and the course of asthma, reflected by changes to prescribing patterns and exacerbation rates.

### Ethics Statement

The General Practice Research Database (GPRD) has a single Multi-Centre Ethics approval for all observational studies using GPRD data (Trent MREC, ref: 05/MRE04/87).

## Methods

The General Practice Research Database (GPRD) contains anonymised longitudinal data collected by general practitioners within UK primary care. [12] Females were identified in the GPRD if they had a pregnancy ending between 1 January 2000 and 31 December 2008 and were aged 11–50 years on the pregnancy end date. All types of pregnancy outcome were captured, including: live births, stillbirths, induced terminations and spontaneous pregnancy losses. An algorithm was used to estimate as accurately as possible the start date of the pregnancy (first day of last menstrual period)[13]–[14] and pregnancies were eligible for inclusion if the female had been registered as a permanently registered patient with the GP for at least the twelve months before, throughout and the six months after the end of pregnancy.

Building on diagnosis code lists developed by Thomas *et al*, [15] females were identified as having asthma if they had at least one recorded asthma diagnosis and they had received at least one prescription for an asthma medicine. Females without a recorded asthma diagnosis who had received six or more prescriptions for asthma medication and who did not have a recorded diagnosis for chronic obstructive pulmonary disease were also included. This was based on a discussion with a respiratory clinician and preliminary work showing that over 85% of all females with an asthma diagnosis in the study cohort had received at least six prescriptions for an asthma medicine. Individuals with a diagnosis of any other chronic respiratory disease (e.g. cystic fibrosis) were excluded.

The final study population consisted of all pregnancies affected by asthma that resulted in a delivery and where asthma medication had been prescribed at least once in the year before, during, or in the six months immediately following pregnancy. Where there were two separate pregnancies in close succession for the same individual and the six months following one pregnancy overlapped with the twelve months before the next, the second pregnancy was excluded. If the start date of the second pregnancy overlapped with the six months following the previous pregnancy event, both pregnancies were excluded.

All prescriptions for asthma medication written by GPs for study participants were identified. This included all short-acting β_2_ agonists (SABAs), inhaled corticosteroids (ICS), long-acting β_2_ agonists (LABAs), compound bronchodilator preparations, cromoglicates, leukotriene receptor antagonists, antimuscarinic bronchodilators and theophylline products. In addition oral corticosteroids that did not appear to have been prescribed for conditions other than asthma were identified. This was done by reviewing all of the medical codes recorded on the same date as the oral corticosteroid prescription. Oral corticosteroid prescriptions where a diagnosis of asthma was recorded on the same day were classified as being asthma-related and those with no indication recorded were identified and included in sensitivity analyses, unless the woman had a diagnosis for an autoimmune disease recorded previously in her record in which case the prescriptions were classified as non-asthma and excluded.

Given the nature of treatment with SABAs for acute symptom relief, no attempt was made to calculate a treatment dose or duration for SABA treatment. Instead, once a woman had received at least one prescription for a SABA it was assumed she had a SABA at her disposal and she was at least on a treatment Step 1 according to the British Thoracic Society (BTS) guidelines on the management for asthma, [16] which are very similar to the GINA guidelines. [17] For the remaining products, the duration of each prescription was calculated by dividing the total number of puffs or tablets in an inhaler or pack by the prescribed daily dosage, taking into account where more than one inhaler or pack was prescribed in a single prescription. Where the quantity or daily dose information had not been recorded, the missing values were imputed based on the value recorded for the nearest prior prescription for the same product. Where this was not available the modal value for that product within the study cohort was taken.

All prescriptions were mapped based on their calculated durations to identify the different treatment combinations individuals were exposed to at different time periods. Where the prescribing information was suggestive of drug switching (e.g. a patient receiving a prescription for a high dose ICS before the end date of a standard dose ICS), the duration of the first product was truncated on the date the second one was received. For oral corticosteroid prescriptions, only those contributing to continuous exposure periods of >60 days duration were included in the mapping. All other oral corticosteroid prescriptions included were taken as evidence of short-term use for the treatment of an acute asthma exacerbation and identified as potentially representing an exacerbation.

**Figure 1:**
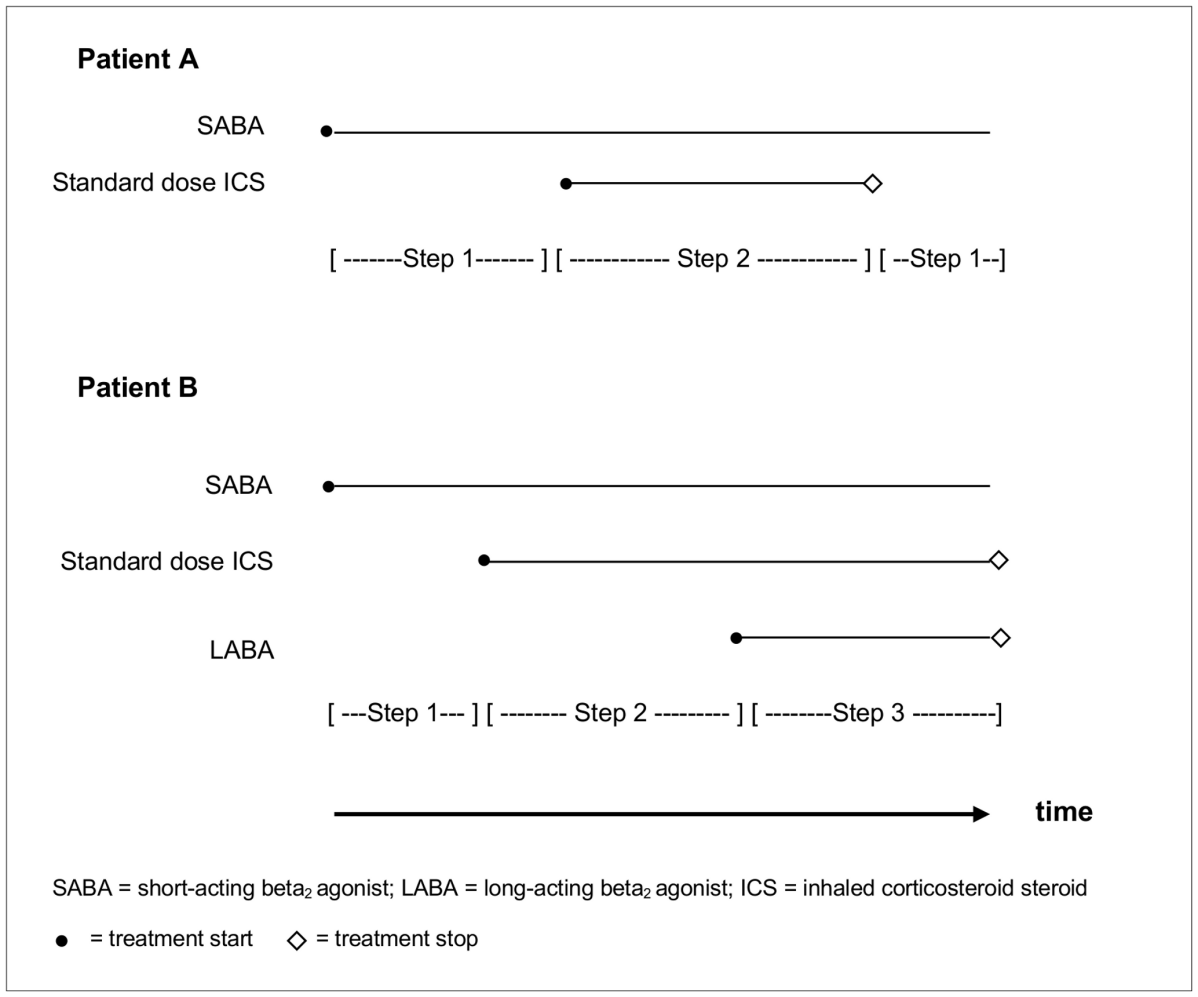
Example scenario for extracts of patients’ mapped prescription records and the allocated corresponding treatment steps.

For each pregnancy, using the BTS guidelines on the management of asthma, this prescription mapping was used to establish which treatment steps females were being prescribed (Figure 1). For product combinations that did not directly translate to a specific treatment step, the most comparable treatment step was assigned (<5% of treatment episodes). Given that within Step 3 of the British guidelines there is considerably more scope for change in treatment intensity than in other treatment steps, two subcategories were created to ensure sensitivity when measuring change in asthma control in people on treatment Step 3. Step 3.3 was created for individuals simultaneously exposed to a SABA, standard dose ICS, LABA and an additional product (e.g. leukotriene receptor) whilst Step 3.6 included those who were on a maximum standard dose ICS in addition to a SABA and LABA, regardless of a prescription for an additional product.

Preliminary review of the prescription mapping and treatment step allocation identified a large proportion of patients who fluctuated between two different treatment steps: 75% of those experiencing a step down in treatment returned to their previous medication within a 2-month period. In most cases this was considered to be the likely result of low persistence with treatment (for example, with a patient not taking the ICS as frequently or in the quantity indicated on the dosage instruction, resulting in it lasting longer with lower average exposure than the calculated assumed duration) rather than evidence of a true improvement and subsequent worsening in asthma control. Therefore, in the prescription mapping any steps down of ≤60 days were ignored where the individual then stepped back up to the same treatment combination they were prescribed previously. Even when steps down that lasted for a duration of ≤60 days were ignored, there was still a large number of women whose combination of asthma medicines and subsequent asthma treatment step changed during a particular 3 month period or pregnancy trimester. To try to account for this, for each individual, an average treatment step value was calculated for each pregnancy trimester and for each of the corresponding time periods in the year before pregnancy (to control for seasonal influences on asthma). This was calculated as
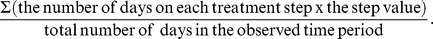



Individuals were categorised into one of three ‘asthma treatment intensity levels’ based on their average treatment step value during each particular time period of interest, with those on an average treatment step≤1 considered to have mild, those on an average treatment step of >1 and ≤2 considered to have moderate, and those on an average treatment step>2 considered to have ‘considerable to severe’ asthma treatment intensity. Whilst the latter category included a wide range of treatment steps, in practice the vast majority within this category was prescribed treatment Step 3.

Individuals were flagged as having an asthma exacerbation each time they had one of the following recorded:

- An asthma exacerbation, asthma attack or asthma diagnosis record on the same date as a hospitalisation or visit to an accident and emergency department, which was classified as a ‘definite’ asthma exacerbation;
- A prescription for short-term oral corticosteroid treatment associated with a record of asthma (but not explicitly coded by the GP as an exacerbation) on the same day, which was classified as a ‘probable’ asthma exacerbation;
- A prescription for short-term oral corticosteroid treatment without any record of the indication for treatment, which was classified as a ‘possible’ asthma exacerbation.

### Analyses

Asthma prevalence in pregnancy was calculated as the number of pregnancies affected by asthma divided by the total number of pregnancies identified. Class-level prescribing patterns of asthma medicines were described in three-month periods for the year leading up to pregnancy, each of the pregnancy trimesters and the six months following pregnancy. Percentages were calculated as a proportion, with the denominator defined as all deliveries where the female had received a prescription for asthma medication in the twelve months before, during or in the six months after pregnancy.

To control for seasonal influences on asthma treatment intensity, the average treatment step in each of the pregnancy trimesters was compared with the corresponding time period in the year before. The percentages of pregnancies in which the average treatment step increased, decreased and remained the same compared with the prior year were calculated for each trimester.

The percentage of pregnancies affected by ‘definite’, ‘probable’, or ‘possible’ exacerbations was calculated separately for each pregnancy trimester and Chi-squared tests were used to compare them with the corresponding time period in the year before. In addition the data was stratified and presented graphically by the patients’ asthma treatment intensity level based on their average treatment step value during each trimester.

A relative risk with 95% confidence intervals was calculated to determine whether females were more likely to experience an asthma exacerbation during pregnancy if they had experienced one in the same time period the year previously compared with those females who had not.

## Results

**Figure 2:**
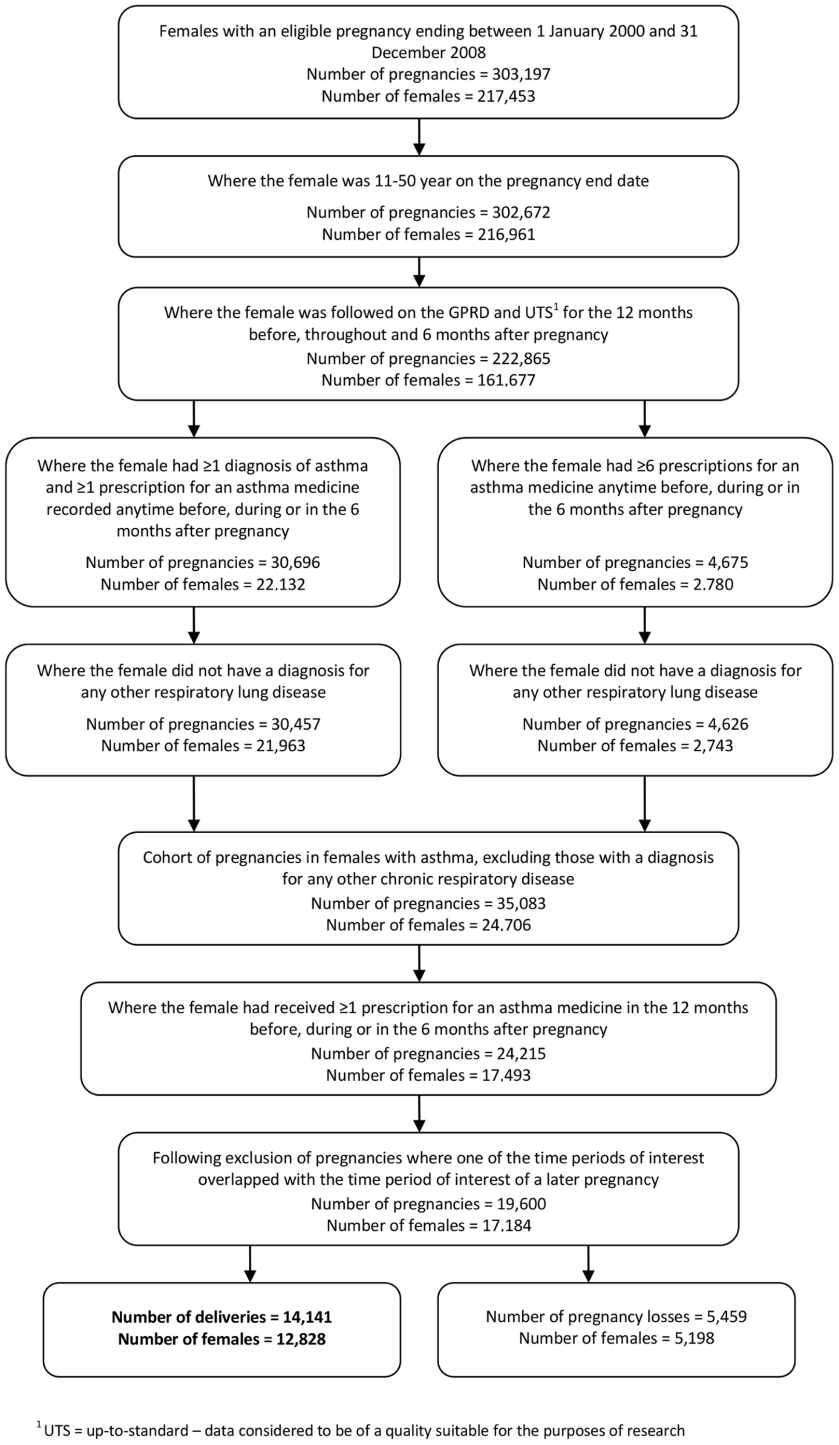
Identifying eligible pregnancies in females with asthma.

From amongst 222,865 pregnancies in females with sufficient follow-up, 19,600 (8.8%) were in 17,184 females with evidence of asthma who had received at least one prescription for an asthma medicine during the time period of interest. Of the 19,600 pregnancies, 14,141 (72.1%) ended in a delivery (Figure 2). The resulting prevalence of asthma affecting pregnancies ending in a delivery was 10.9% (CI_95_ 10.7–11.0). Narrowing this down to deliveries where prescriptions for asthma medicines were issued during the actual pregnancy period as opposed to before, during or after pregnancy, the prevalence became 8.3% (CI_95_ 8.1–8.4). Characteristics, including maternal age and smoking status, for females included in the final study population are given in Tables 1 and 2.

**Table 1:** Population characteristics for the final asthma-pregnancy cohort.

Characteristic	Subcategory	N
Number of pregnancy outcomes		19,600
Distinct number of females		17,184
Type of pregnancy outcome	Deliveries	14,141
	Pregnancy losses	5,459
Mean age at pregnancy outcome(years: (SD))	All pregnancies	29.7 (6.6)
	Deliveries	30.1 (6.1)
	Pregnancy losses	28.8 (7.8)

**Table 2:** Population characteristics for the cohort of pregnancies that resulted in a delivery.

Characteristic	Subcategory	N	(%)
Age at delivery (years)	<20	675	4.8
	20–24	2,231	15.8
	25–29	3,385	23.9
	30–34	4,242	30.0
	35–39	2,852	20.2
	40+	756	5.3
Smoking status*	Non-smoker	7,228	51.1
	Current smoker	4,447	31.4
	Ex-smoker	2,411	17.0
	Unknown	55	0.4
Alcohol drinking status*	Teetotal	1,940	13.7
	Drinks alcohol	9,638	68.2
	Heavy drinker	152	1.1
	Ex-drinker	516	3.6
	Unknown	1,895	13.4
Body mass index*	<20	1,286	9.1
	20–24	4,853	34.3
	25–29	2,754	19.5
	30–34	1,204	8.5
	>34	834	5.9
	Unknown	3,210	22.7
Socioeconomic status(practice level)	Quintile 1– leastdeprived	2,693	19.0
	Quintile 2	2,398	17.0
	Quintile 3	2,862	20.2
	Quintile 4	2,776	19.6
	Quintile 5	3,412	24.1

* Nearest to pregnancy start date.

In 68.4% of pregnancies among women with evidence of asthma ending in a delivery, a prescription was issued for a SABA. Prescriptions were issued for an ICS, LABA, or a combination product in 41.2%, 4.9% and 8.9% of pregnancies respectively. A further 0.1% who were not prescribed a SABA, ICS, or LABA-containing medicine received a prescription for an alternative asthma therapy (e.g. leukotriene receptor antagonist, antimuscarinic bronchodilator, theophylline, or cromoglicate) during pregnancy. Salbutamol was the most commonly prescribed SABA whilst salmeterol was the LABA that individuals had received the most. Over 80% of all prescriptions for the ICS-class were for beclometasone formulations, whereas Seretide® (fluticasone and salmeterol) was the most commonly prescribed combination product. Table S1 shows a breakdown of prescribing during each of the time periods.

In 45.6% of pregnancies, asthma was managed only with BTS treatment Step 1. Females received asthma Step 2 therapy at some point during pregnancy in 35.5% of pregnancies, whilst in 16.4% of pregnancies, females received asthma management from Step 3; 6.5% received Step 4 and 0.04% received Step 5 management at some point during pregnancy. When categorising pregnancies into asthma treatment intensity levels based on average treatment step values, 50.2% were categorised as ‘mild’ (average step≤1), 37.7% as ‘moderate’ (average step>1 and ≤2) and 12.1% as ‘considerable to severe’ (average step>2).

**Figure 3:**
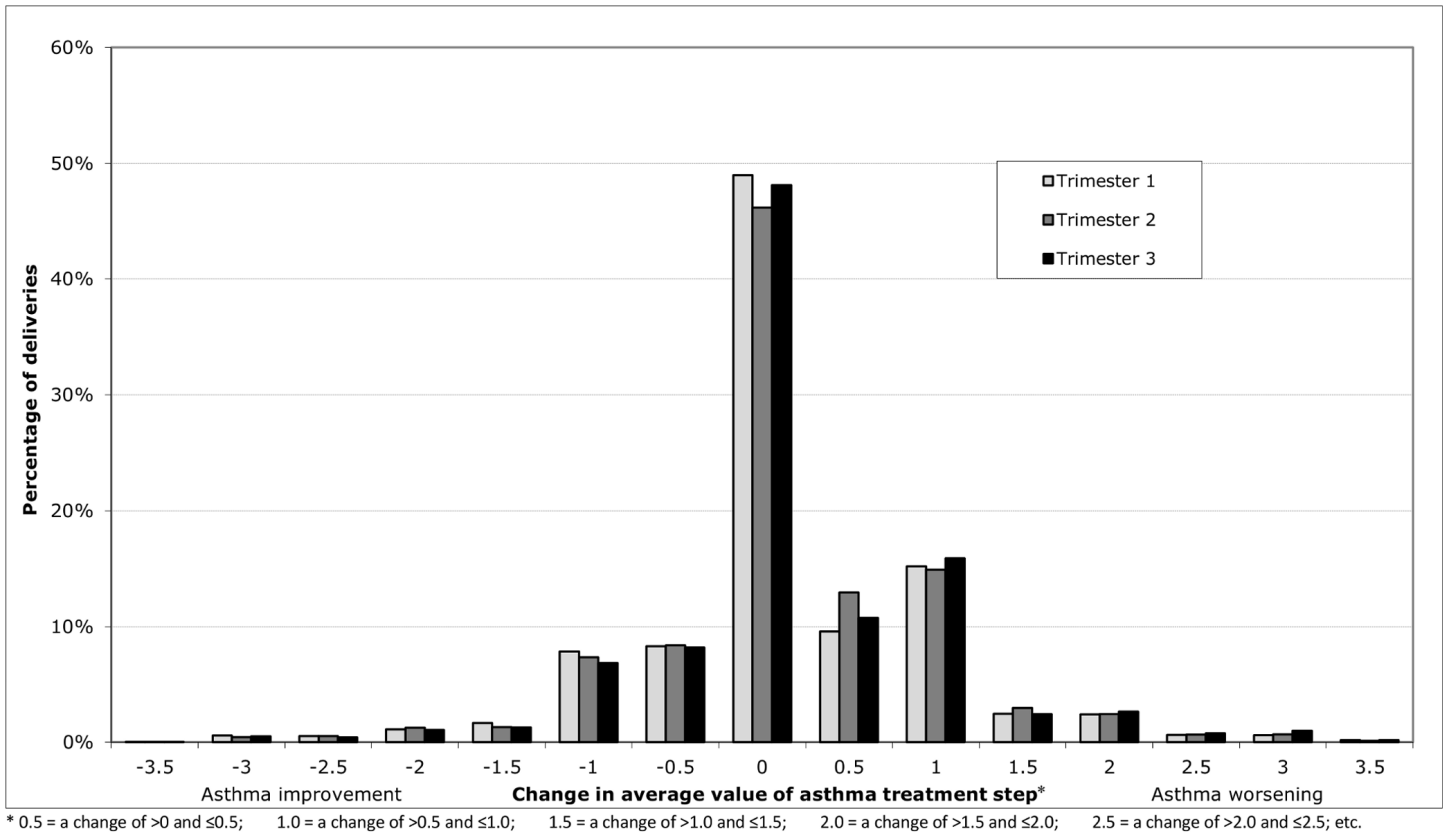
Change in average British Thoracic Society (BTS) asthma treatment step. The percentage of deliveries in which the average BTS asthma treatment step increased, decreased or remained unchanged compared with the calendar period 12 months prior stratified by pregnancy trimester.

Figure 3 shows the percentage of deliveries where the female’s average asthma BTS treatment step during each pregnancy trimester increased, decreased or remained the same compared with the same time period the year before. When evaluating changes in treatment intensity over the entire pregnancy period, in 29.4% of pregnancies treatment intensity was the same as in the year before whereas treatment intensity increased in 45.0% and decreased in 25.6% of pregnancies. During the overall pregnancy period 5.5% of females experienced an increase of more than one step change in their average treatment step and 2.1% experienced a decrease of greater than one. No pregnancy trimester stood out as being particularly associated with an increased or decreased frequency of treatment changes; in each individual trimester, for about a third of all women their asthma management increased by some amount (mostly <1 step change) whereas for about a fifth, treatment intensity decreased (also mostly by <1 step change).

From Table 3 it also becomes clear that for many women whose treatment intensity changed, changes happened more than once. For instance, some women who stepped up in treatment in trimester one, also stepped down in trimester two, to step up again in trimester three. This explains why the percentages of changes for pregnancy overall differed from those for the individual pregnancy trimesters.

**Table 3:** The number and percentage of deliveries where the female’s average BTS asthma treatment step increased, decreased or remained the same, when compared with the same calendar time in the year before pregnancy, stratified by pregnancy trimester.

Change in average treatmentstep value^1^	Decreased by>1.0–4.0		Decreased by>0.5–1.0		Decreased by>0.0–0.5		Decreased byany amount		Remainedthe same		Increased byany amount		Increased by>0.0–0.5		Increased by>0.5–1.0		Increased by>1.0–4.0	
	N	(%)	N	(%)	N	(%)	N	(%)	N	(%)	N	(%)	N	(%)	N	(%)	N	(%)
**Trimester 1** 2	561	(4.0)	1081	(7.6)	1106	(7.8)	2748	(19.4)	7007	(49.6)	4386	(31.0)	1322	(9.3)	2154	(15.2)	910	(6.4)
**Trimester 2** 2	492	(3.5)	916	(6.5)	1361	(9.6)	2769	(19.6)	6434	(45.5)	4938	(34.9)	1844	(13.0)	2133	(15.1)	961	(6.8)
**Trimester 3** 3	464	(3.3)	927	(6.6)	1130	(8.0)	2521	(17.9)	6868	(48.7)	4708	(33.4)	1501	(10.6)	2238	(15.9)	969	(6.9)
**Pregnancy overall** 3	293	(2.1)	639	(4.5)	2686	(19.0)	3618	(25.6)	4160	(29.4)	6363	(45.0)	3654	(25.8)	1929	(13.6)	780	(5.5)

1 a decrease in average treatment step was considered to be indicative of an improvement in asthma control whilst an increase in average treatment step was considered to represent a worsening in asthma control.

2 N = 14,141.

3 N = 14,097 as a small number of deliveries were very premature and born at the very end of the second trimester and therefore did not contribute to the third trimester.

When we examined the entire pregnancy period and focused on ‘definite’ exacerbations, 3.3% of pregnancies were affected by an exacerbation whereas when ‘definite’, ‘probable’ and ‘possible’ exacerbation definitions were applied, 6.0% of pregnancies had evidence of an asthma exacerbation occurrence. The percentage of pregnancies with at least one asthma exacerbation increased with the level of asthma treatment intensity. The percentage of pregnancies affected by at least one exacerbation was 1.0% in individuals whose asthma treatment intensity was classified as ‘mild’, 4.8% for ‘moderate’ and 8.0% in those classified as having ‘considerable to severe’ asthma.

**Figure 4:**
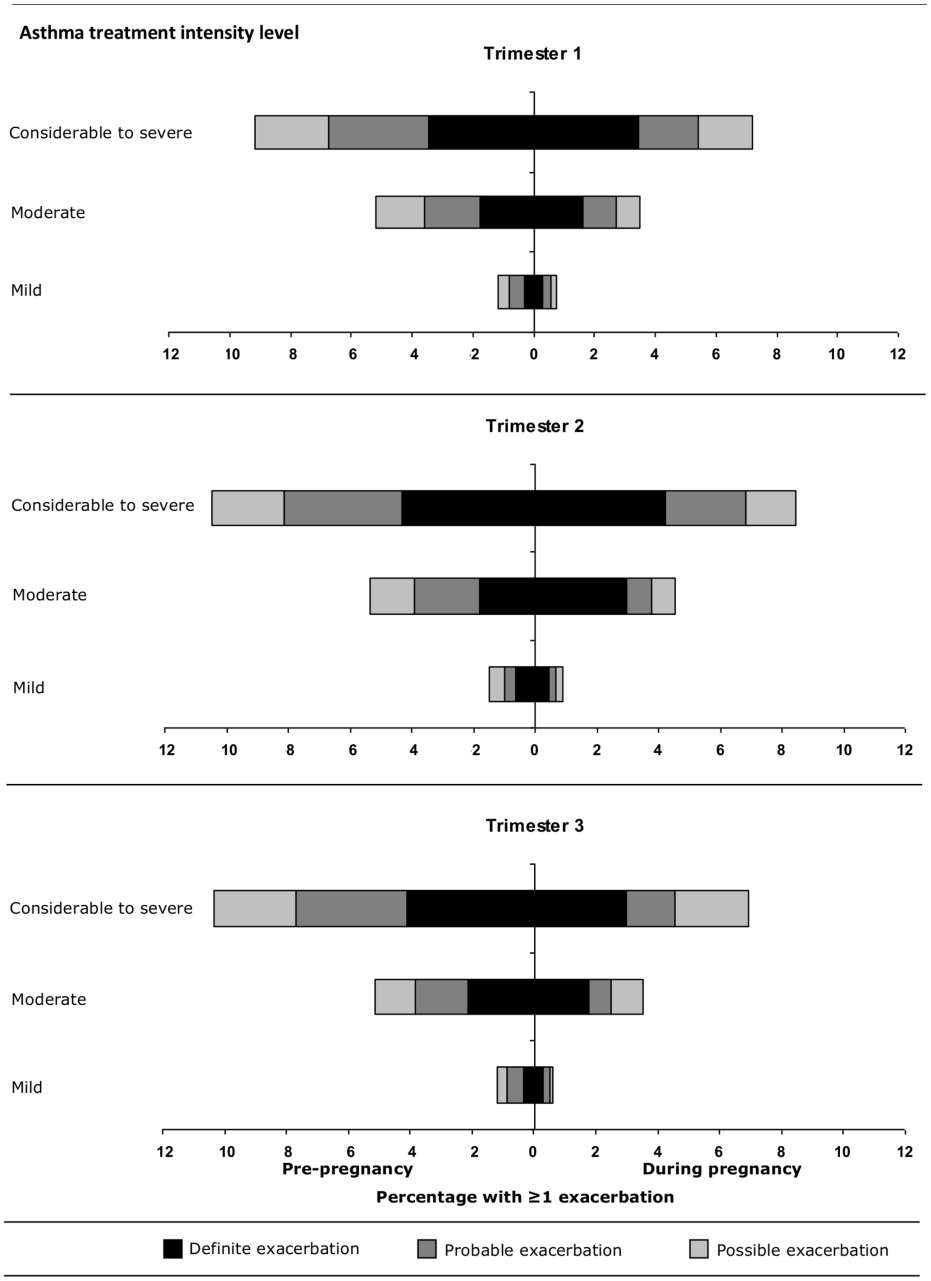
Exacerbations during pregnancy. Stacked bars showing the percentage of deliveries where the mother had ≥1 exacerbation during pregnancy (right hand side) and in the corresponding time period the year before pregnancy (left hand side) stratified by pregnancy trimester and asthma treatment intensity level.^*^ *Asthma treatment intensity level: mild = average step≤1, moderate = average step>1 and ≤2, considerable to severe = average step>2 Definite exacerbation = a medical code for an asthma exacerbation or asthma attack or an asthma diagnosis code recorded on the same date as a hospitalisation or accident and emergency visit; probable exacerbation = a prescription for short-term oral corticosteroid treatment with a record of asthma (but not explicitly an exacerbation) on the same date the prescription was issued; possible exacerbation = a prescription for short-term oral corticosteroid treatment with no record of the indication for treatment.

Figure 4 depicts the difference in exacerbation risk in each pregnancy trimester compared with the risk a year earlier, stratified by asthma treatment intensity level and graphically presented by the degree of certainty as to whether the medical record signified a true asthma exacerbation. Considering ‘definite’ exacerbations alone (defined by asthma exacerbation-related healthcare utilization), for instance, in the second pregnancy trimester 3.0% of women considered to have moderate asthma (an average BTS treatment step>1 and ≤2) experienced a ‘definite’ exacerbation compared with 1.8% in the same period in the calendar year before pregnancy. Based on the ‘definite’ exacerbations alone, with the exception of the prior example, no differences in the risk of exacerbation were observed associated with pregnancy compared with the same calendar period a year earlier (Figure 4). A different pattern emerged in the analyses where ‘probable’ (defined as short-term oral corticosteroid prescribed at an asthma-related GP visit), as well as those where ‘probable’ and ‘possible’ (short-term oral corticosteroid prescription not associated with an asthma-related GP visit) definitions of asthma exacerbations were added: in these analyses, across all trimesters and all levels of asthma treatment intensity, the proportion of women experiencing any exacerbation appeared to be considerably lower during pregnancy relative to the year before pregnancy. Data from Figure 4 also suggest a shift in the way exacerbations were recorded during pregnancy compared with before: during pregnancy, proportionally more potential exacerbations were classified as ‘definite’ and fewer were ‘probable’, whereas the proportion of ‘possible’ exacerbations remained roughly the same. An analysis on this basis of the frequency of ‘definite’ or ‘probable’ defined exacerbations revealed an overall reduction in exacerbation rates from 5.9% to 4.8% associated with pregnancy; a reduction from 2.0% to 1.6% (p = 0.005) in the first trimester, no change in the second (p = 0.20), and a reduction from 2.3% to 1.5% (p<0.001) in the third trimester.

Overall, for all levels of asthma treatment intensity and for all diagnostic certainty levels, females were more likely to experience an asthma exacerbation during pregnancy if they had experienced one in the same time period the year before pregnancy compared with those who had not (RR = 5.7, 95% CI 4.5–7.2).

## Discussion

In this study based on longitudinal electronic medical records with linked prescription data, asthma affected between 8.3–10.9% of pregnancies, compared to 3–14% reported elsewhere. [1]–[4] In the vast majority of asthma-affected pregnancies identified, treatment during pregnancy was confined to SABA only (45.6%) or inhaled corticosteroid-containing products (30.9%), whereas about a quarter of pregnancies had additional treatment prescribed. Acute asthma exacerbations requiring medical intervention occurred in approximately 5% of pregnancies, which varied depending on underlying disease activity and the level of diagnostic certainty considered acceptable. In nearly one-in-three pregnancies, asthma treatment remained the same throughout pregnancy compared to the same time period the year before, whereas in nearly half of pregnancies, treatment intensity was increased by some amount.

To our knowledge this is the first comprehensive, longitudinal population-based study in the past two decades on this subject. It is also one of the largest to evaluate the association between pregnancy and the course of asthma and the first to analyse prescribing of asthma management steps in pregnancy. By including data from the 12 month period leading up to each pregnancy, we were able to control for the influence of seasonality on disease activity in asthma.

In this study, no change in average treatment step associated with pregnancy was observed in 29.4% of pregnancies, whereas treatment intensity was reduced in 25.6% (as indicated by lower average treatment steps) and increased in 45.0% compared with the year before pregnancy. This compares reasonably well with the authoritative study by Schatz *et al* from 1988, who used women’s self-reported data collected in daily diaries and found asthma control was not affected by pregnancy in 33% of 330 women whereas it improved in 28% and worsened in 35%. [9] The results differ, however, with those reported by Louik *et al*, where based on an interview up to six months following pregnancy 53.0% of women reported no overall change in their symptoms during pregnancy, 24.6% reported an overall improvement and 22.4% reported an overall worsening. [2] The study by Louik *et al*. did not allow for combinations of worsening and improvement, which facilitated the identification of associated risk factors. In our study, however, we allowed for a fluctuation between worsening and improvement throughout pregnancy. In general, the heterogeneous methods used between studies evaluating the impact of pregnancy on the course of asthma and the subjective nature and different definitions of asthma symptoms and control make it difficult to compare studies directly.

The increases and decreases observed in our study may to some extent indicate worsening and improvement of asthma activity and/or asthma control although it is accepted that some women may choose to stop taking their asthma medicines when they become aware they are pregnant and others may choose to become more compliant during pregnancy in an attempt to reduce the likelihood of an exacerbation of their asthma. In our study, changes in average asthma treatment step were observed during all pregnancy trimesters and the consistency across trimesters suggests there was not one time period during pregnancy when changes were most likely to occur. The fact that 19.4% of females experienced some reduction in average treatment step during the first trimester could support the theory that a number of women stop taking their medicines once they realise they are pregnant, however, the fact that a similar percentage experienced some reduction during the second and third trimester suggests it may be more than this and could also be indicative of improvement in asthma symptoms. Similarly the large percentage of females experiencing an increase in average treatment step during the first trimester may imply low-persistence and a change during pregnancy to become more compliant but equally as this is present across all trimesters it may also suggest a worsening of asthma symptoms and a need for greater control in a subset of pregnancies. Unlike the studies by Schatz *et al* [9] and Louik *et al*, [2] determination of disease activity in this study was based on analysis of retrospective, routinely collected prescription data via general practitioner recording and no information was available direct from the females or their clinicians. Information regarding symptoms occurring during sleep and whether GP visits were scheduled or not is not recorded consistently in the GPRD. In this study therefore, conclusions regarding asthma activity improving, worsening, or remaining the same are inferred from treatment patterns rather than confirmed by females or their doctors. Also, as a consequence of the nature of asthma management, various assumptions had to be made regarding the timing and duration of exposure to asthma medicines. Although these assumptions were based on experience in clinical practice as well as observations of treatment patterns, they will have resulted in some misclassification of exposure to different asthma management steps. In addition the algorithm used to carry out the prescription mapping and assign patients to asthma treatment steps has not been validated. However, various sensitivity analyses did not materially alter the results regarding the proportion of pregnancies in which asthma treatment intensity appeared to have improved, worsened, or remained the same. The creation of an average treatment step was beneficial for enabling comparisons to be made over a three-month time period with the previous year, however, it is possible that some of the small changes identified (i.e. <0.5 treatment step) may not be clinically relevant. It is also possible that what was identified as ‘worsening’ in some cases merely reflected better disease management of asthma whereas disease activity actually remained the same and what was identified as ‘improving’ may have in some cases reflected low persistence and women opting not to take their asthma medicines as instructed rather than a true improvement in asthma activity. Of those females who appeared to be ‘stepping up’ and ‘stepping down’ regularly between different treatment steps, 75% consistently stepped down to treatment without ICS, only to step up again to the previous treatment step that included the ICS within less than a 30- or 60-day period not covered by an ICS prescription. Low persistence to ICS treatment therefore appeared to be common, and we took this into account when developing our algorithms for the determination of treatment steps. Using electronic health care records alone, it is not possible to establish whether this ‘low persistence’ reflected females’ capacity to self-titrate their treatment, whether it reflected poor asthma management on the part of the female or whether it resulted from the decisions made and advice given by the GP in terms of how often and in what quantity they should use their ICS. In this study 31.4% of deliveries were to females who smoked, which is in agreement with the findings of Louik *et al.* [2] and conflicts with existing guidelines of asthma triggers where smoking is referenced along with other teratogenic exposures to avoid during pregnancy. [18]Depending on the level of diagnostic certainty from database definitions, between 3.3% and 6.0% of pregnancies ending in a delivery in women with asthma were affected by an asthma exacerbation. This percentage increased directly with the calculated asthma treatment intensity level, the percentage of deliveries where the female experienced at least one asthma exacerbation being 1.0–2.1% for those with ‘mild’, 4.8–8.4% for those with ‘moderate’ and 8.0–15.9% for those with ‘considerable to severe’ asthma treatment intensity respectively. Overall, there was no suggestion of pregnancy being associated with an increased risk of asthma exacerbation: depending on the degree of diagnostic certainty, pregnancy appeared to be associated with no change (‘definite’ exacerbations only) or a reduction (including ‘probable’, with or without ‘possible’, exacerbations) in the frequency of asthma exacerbations compared with the frequency in the same season one year before the pregnancy. This reduction in exacerbations may reflect an improvement in disease severity during pregnancy or greater asthma control in women who prior to pregnancy suffered exacerbations as a result of being non-compliant with their treatment. Alternatively, the observed reduction may be an artefact of the database: during or following an exacerbation pregnant women may be more likely to seek care from a hospital or accident and emergency department and these visits and associated prescriptions are not always captured in GP records. In general, even with the restrictions imposed in this study, oral corticosteroid prescriptions may be a poor indicator of exacerbations, especially if GPs are less likely to prescribe pregnant women a supply of oral corticosteroids to stockpile/keep in case of a future exacerbation or if GPs are less likely to prescribe oral steroids in general to women who are pregnant even following an exacerbation, as has been observed in an emergency department in the USA. [19].

In conclusion, 8.3% of females with a pregnancy resulting in a delivery received a prescription for some form of asthma medicine during pregnancy and 31.4% of these pregnancies were to females who smoked. Changes in asthma treatment intensity were common and occurred at all stages of pregnancy, however, the extent to which these changes reflect true changes in asthma activity/severity is unknown. We observed a high frequency of what appeared to be low persistence with maintenance treatment. At about 5% occurrence, an asthma exacerbation was not uncommon and the frequency of asthma exacerbations increased with increasing asthma treatment intensity. Given that poorly controlled asthma increases the risk of preterm labour, pre-eclampsia and low birth weight in the offspring, [20] this study highlights the need to discuss asthma management and reinforce the importance of appropriate disease management, including smoking cessation, for females with asthma who become pregnant.

## Supporting Information

Table S1 Number and percentage of deliveries where the female received a prescription for an asthma medicine during one of the time periods of interest.
(DOCX)
