# Supplementary material for: Asthma Management in Pregnancy
Source: PLoS One. 2013 Apr 4;8(4):e60247. doi: 10.1371/journal.pone.0060247 (PMC3617219; doi:10.1371/journal.pone.0060247)
Supplement: Table S1 — Number and percentage of deliveries where the female received a prescription for an asthma medicine during one of the time periods of interest. (DOCX) [file pone.0060247.s001.docx]

**Table S1** Number and percentage of deliveries where the female received a prescription for an anti-asthma medicine during one of the time periods of interest (N= 14,141)

| **Class of asthma medicine** | **Asthma medicine** | **12-9m before** | **9-6m before** | **6-4m before** | **3-0m before** | **1^st^ Tri^a^** | **2^nd^ Tri^b^** | **3^rd^ Tri^c^** | **0-3m after** | **4-6m after** | **During pregnancy** | | **During any of the time periods** | |
| --- | --- | --- | --- | --- | --- | --- | --- | --- | --- | --- | --- | --- | --- | --- |
| **Short acting β_2_ agonist** | Salbutamol | 4,770 | 4,689 | 4,672 | 4,626 | 4,506 | 6,016 | 5,000 | 4,645 | 4,800 | **8,974** | **(63.5%)** | **12,749** | **(90.2%)** |
|  | Terbutaline | 427 | 399 | 373 | 376 | 396 | 479 | 424 | 367 | 359 | **762** | **(5.4%)** | **1,156** | **(8.2%)** |
|  | Orciprenaline | 11 | 8 | 11 | 12 | 8 | 6 | 5 | 4 | 7 | **19** | **(0.1%)** | **67** | **(0.5%)** |
|  | Fenoterol | 0 | 0 | 0 | 0 | 0 | 0 | 0 | 0 | 0 | **--** | **--** | **--** | **--** |
|  | Isoprenaline | 0 | 0 | 0 | 0 | 0 | 0 | 0 | 0 | 0 | **--** | **--** | **--** | **--** |
|  | Reproterol | 0 | 0 | 0 | 0 | 0 | 0 | 0 | 0 | 0 | **--** | **--** | **--** | **--** |
|  | Rimiterol | 0 | 0 | 0 | 0 | 0 | 0 | 0 | 0 | 0 | **--** | **--** | **--** | **--** |
|  | **Total distinct pregnancies** | **5,177** | **5,073** | **5,033** | **4,991** | **4,890** | **6,480** | **5,411** | **4,990** | **5,148** | **9,670** | **(68.4%)** | **13,646** | **(96.5%)** |
| **Long acting β_2_ agonist** | Salmeterol | 415 | 432 | 395 | 390 | 361 | 440 | 399 | 352 | 375 | **643** | **(4.5%)** | **1,035** | **(7.3%)** |
|  | Formoterol | 39 | 46 | 36 | 37 | 33 | 39 | 34 | 34 | 37 | **55** | **(0.4%)** | **89** | **(0.6%)** |
|  | Bambuterol | 0 | 0 | 0 | 0 | 1 | 0 | 0 | 2 | 1 | **1** | **(<0.01%** | **2** | **(0.01%)** |
|  | Eformoterol | 0 | 0 | 0 | 0 | 0 | 0 | 0 | 0 | 0 | **--** | **--** | **--** | **--** |
|  | **Total distinct pregnancies** | **454** | **478** | **431** | **427** | **393** | **479** | **432** | **388** | **413** | **695** | **(4.9%)** | **1,117** | **(7.9%)** |
| **Inhaled corticosteroids** | Beclometasone | 2,391 | 2,334 | 2,375 | 2,227 | 2,194 | 3,052 | 2,580 | 2,220 | 2,313 | **4,791** | **(33.9%)** | **7,314** | **(51.7%)** |
|  | Budesonide | 334 | 319 | 293 | 294 | 277 | 378 | 333 | 285 | 280 | **587** | **(4.2%)** | **905** | **(6.4%)** |
|  | Fluticasone propionate | 335 | 316 | 291 | 309 | 298 | 344 | 312 | 269 | 273 | **498** | **(3.5%)** | **724** | **(5.1%)** |
|  | Ciclesonide | 2 | 1 | 2 | 2 | 3 | 2 | 0 | 4 | 3 | **5** | **(0.04%)** | **12** | **(0.1%)** |
|  | Mometasone | 8 | 10 | 9 | 6 | 6 | 5 | 5 | 8 | 8 | **8** | **(0.06%)** | **23** | **(0.2%)** |
|  | **Total distinct pregnancies** | **3,051** | **2,972** | **2,949** | **2,830** | **2,764** | **3,764** | **3,215** | **2,772** | **2,868** | **5,824** | **(41.2%)** | **8,702** | **(61.5%)** |
| **Combination products** | Salbutamol + beclometasone | 7 | 6 | 3 | 3 | 1 | 5 | 0 | 3 | 3 | **6** | **(0.04%)** | **18** | **(0.1%)** |
|  | Formoterol + beclometasone | 0 | 0 | 0 | 0 | 0 | 0 | 0 | 0 | 0 | **--** | **--** | **--** | **--** |
|  | Formoterol + budesonide | 145 | 153 | 172 | 183 | 195 | 236 | 222 | 220 | 245 | **326** | **(2.3%)** | **468** | **(3.3%)** |
|  | Salmeterol + fluticasone prop | 491 | 558 | 601 | 626 | 652 | 712 | 717 | 673 | 729 | **958** | **(6.8%)** | **1,269** | **(9.0%)** |
|  | Salbutamol + sodium cromo. | 0 | 0 | 0 | 0 | 0 | 0 | 0 | 0 | 1 | **0** | **(0.0%)** | **1** | **(<0.01%** |
|  | Salbutamol + Ipratropium | 13 | 8 | 15 | 14 | 14 | 19 | 15 | 12 | 12 | **28** | **(0.2%)** | **47** | **(0.3%)** |
|  | Fenoterol + Ipratropium | 5 | 5 | 6 | 6 | 5 | 6 | 4 | 4 | 4 | **6** | **(0.04%)** | **7** | **(0.05%)** |
|  | Isoprenaline + sodium cromo. | 0 | 0 | 0 | 0 | 0 | 0 | 0 | 0 | 0 | **--** | **--** | **--** | **--** |
|  | **Total distinct pregnancies** | **646** | **713** | **775** | **813** | **847** | **955** | **938** | **895** | **976** | **1,264** | **(8.9%)** | **1,694** | **12.0%)** |

^a^ Trimester 1 = weeks 1-12, ^b^ Trimester 2 = Weeks 13-27, ^c^ Trimester 3 = Weeks 28 to the end of pregnancy

**Table S1 continued**.

| **Class of asthma medicine** | **Asthma medicine** | **12-9m before** | **9-6m before** | **6-4m before** | **3-0m before** | **1^st^ Tri^a^** | **2^nd^ Tri^b^** | **3^rd^ Tri^c^** | **0-3m after** | **4-6m after** | **During pregnancy** | | **During any of the time periods** | |
| --- | --- | --- | --- | --- | --- | --- | --- | --- | --- | --- | --- | --- | --- | --- |
| **Cromoglicate & leukotriene receptor antagonists** | Montelukast | 85 | 92 | 94 | 96 | 77 | 35 | 29 | 60 | 74 | **83** | **(0.6%)** | **210** | **(1.5%)** |
|  | Zafirlukast | 4 | 6 | 5 | 6 | 6 | 3 | 3 | 5 | 5 | **7** | **(0.05%)** | **8** | **(0.1%)** |
|  | Sodium cromoglicate | 13 | 6 | 6 | 7 | 9 | 9 | 6 | 10 | 10 | **19** | **(0.1%)** | **28** | **(0.2%)** |
|  | Nedocromil sodium | 1 | 2 | 1 | 1 | 1 | 1 | 0 | 1 | 1 | **2** | **(0.01%)** | **5** | **(0.04%)** |
|  | **Total distinct pregnancies** | **97** | **106** | **105** | **110** | **93** | **48** | **38** | **75** | **89** | **111** | **(0.8%)** | **251** | **(1.8%)** |
| **Antimuscarinic bronchodilators** | Ipratropium | 27 | 27 | 37 | 33 | 30 | 39 | 32 | 24 | 22 | **65** | **(0.5%)** | **117** | **(0.8%)** |
|  | Tiotropium | 3 | 1 | 2 | 2 | 3 | 1 | 2 | 2 | 4 | **3** | **(0.02%)** | **6** | **(0.04%)** |
|  | Oxitropium | 3 | 2 | 2 | 2 | 3 | 4 | 3 | 2 | 3 | **5** | **(0.04%)** | **6** | **(0.04%)** |
|  | **Total distinct pregnancies** | **32** | **29** | **40** | **36** | **36** | **44** | **37** | **28** | **29** | **72** | **(0.9%)** | **125** | **(0.9%)** |
| **Theophylline** | Theophylline | 31 | 34 | 29 | 27 | 27 | 30 | 20 | 23 | 23 | **39** | **(0.3%)** | **65** | **(0.5%)** |
|  | Aminophylline | 21 | 16 | 14 | 17 | 13 | 16 | 15 | 16 | 15 | **16** | **(0.1%)** | **31** | **(0.2%)** |
|  | **Total distinct pregnancies** | **52** | **50** | **43** | **44** | **40** | **46** | **35** | **39** | **36** | **55** | **(0.4%)** | **94** | **(0.5%)** |
| **Oral corticosteroids^*^** | Betamethasone | 0 | 0 | 0 | 0 | 0 | 0 | 0 | 0 | 0 | -- | -- | **--** | **--** |
|  | Dexamethasone | 0 | 0 | 0 | 0 | 1 | 0 | 0 | 0 | 0 | **1** | **(<0.01%** | **1** | **<0.01%)** |
|  | Hydrocortisone | 0 | 0 | 0 | 0 | 0 | 0 | 0 | 0 | 0 | **--** | **--** | **--** | **--** |
|  | Prednisolone | 304 | 285 | 294 | 290 | 178 | 195 | 170 | 188 | 242 | **464** | **(3.3%)** | **1,541** | **(10.9%)** |
|  | **Total distinct pregnancies** | **304** | **285** | **294** | **290** | **179** | **195** | **170** | **188** | **242** | **464** | **(3.3%)** | **1,541** | **(10.9%)** |

^a^ Trimester 1 = weeks 1-12, ^b^ Trimester 2 = Weeks 13-27, ^c^ Trimester 3 = Weeks 28 to the end of pregnancy

^*^ assumed to have been prescribed for asthma
